# Digital signatures of fatigue and wearing-off symptoms in patients with multiple sclerosis under natalizumab

**DOI:** 10.1016/j.neurot.2026.e01075

**Published:** 2026-09-18

**Authors:** Lars Masanneck, Elaine Trautmann, Judith von Pavel, Ramona Hagler, Marius Vach, Patricia Kirschner, Jan Voth, Melanie Schuette, Pascal Benkert, Jens Kuhle, Jörn Kaufmann, Marie Deffner, Nicholas Schwab, Steffen Pfeuffer, Sven G. Meuth, Marc Pawlitzki

**Affiliations:** a Department of Neurology, Medical Faculty, Heinrich-Heine-University Düsseldorf, Moorenstraße 5, Düsseldorf, 40225, Germany; b Hasso Plattner Institute, University of Potsdam, Potsdam, Germany; c Department of Diagnostic and Interventional Radiology, Medical Faculty and University Hospital Düsseldorf, Heinrich-Heine-University Düsseldorf, Moorenstraße 5, Düsseldorf, 40225, Germany; d Multiple Sclerosis Centre and Research Center for Clinical Neuroimmunology and Neuroscience (RC2NB), Departments of Biomedicine and Clinical Research, University Hospital and University of Basel, Basel, Switzerland; e Department of Neurology, Otto-von-Guericke University, Leipziger Straße 44, Magdeburg, 39120, Germany; f Department of Neurology, University Hospital Muenster, Muenster, Germany; g Department of Neurology, University Hospital Giessen and Marburg, Justus-Liebig-University Giessen, Giessen, Germany

**Keywords:** Multiple sclerosis, sNfL, MRI, Wearables, Wearing-off symptoms, Natalizumab

## Abstract

Wearing-off symptoms (WoS) during natalizumab (NTZ) treatment are frequently interpreted as subclinical disease activity in patients with multiple sclerosis (PwMS), but their significance remains uncertain. In the prospective single-center SATURATE study (NCT05701423), we investigated whether WoS were associated with inflammatory or neurodegenerative damage and whether wearable monitoring captures related motor changes. Thirty-four NTZ-treated participants were enrolled; 28 completed 6-month follow-up and 21 provided analyzable smartwatch data. Clinical assessments were performed at baseline, month 3, and month 6; serum NTZ, soluble vascular cell adhesion molecule-1, neurofilament light chain, glial fibrillary acidic protein, and diffusion-weighted magnetic resonance imaging were assessed at baseline and month 6. Before each NTZ administration, participants completed a WoS questionnaire. WoS occurred in 19/28 analyzed participants (67.9%) and 63/177 of observed questionnaires (35.6%). WoS rate was associated with fatigue, but not with dosing interval, diffusion changes, or serum biomarkers. The adjusted subcutaneous-versus-intravenous coefficient was −0.296 (HC3-robust 95% CI, −0.649 to 0.056; p = 0.100; conventional OLS 95% CI, −0.575 to −0.018; p = 0.038). Wearable data showed a subtle end-of-cycle decline in afternoon steps (within-subject z-score difference, −0.130; 95% CI, −0.229 to −0.004; Holm-adjusted p = 0.015). Dynamic activity features, including morning-to-afternoon step ratios and reduced peak step variability, correlated with fatigue scores and WoS rate. These exploratory findings indicate that, in this clinically stable cohort, WoS under NTZ do not reflect ongoing inflammatory or neurodegenerative disease activity, but rather align with fatigue-related motor changes detectable by high-adherence passive wearables. Dynamic wearable activity metrics warrant evaluation as digital endpoints for fatigue-focused interventions and clinical care.

## Introduction

Following nearly two decades of successful clinical use in multiple sclerosis (MS), natalizumab (NTZ) has also been approved for both subcutaneous (SC) administration and extended interval dosing (EID) [1,2]. SC NTZ shows comparable pharmacokinetics and clinical outcomes to intravenous (IV) NTZ, and EID has been associated with reduced PML risk and maintained clinical efficacy [[3], [4], [5]].

Despite these advances in treatment optimization, a clinically relevant proportion of patients experience so-called “wearing-off symptoms” (WoS) [6,7]. These are typically characterized by increasing fatigue and, in some cases, a temporary worsening of pre-existing neurological deficits toward the end of the infusion interval [6].

Although the exact mechanisms of WoS remain unclear [8,9], a dose effect and resulting wearing-off phenomenon appear biologically plausible, given that NTZ blocks leukocyte access to the brain and spinal cord [9]. *Bringeland* et al. suggest that VLA-4 (very late antigen-4) receptor desaturation may contribute to WoS and could be linked to higher body weight or other factors promoting accelerated NTZ clearance [10].

More importantly, it remains unclear whether these symptoms merely reflect pharmacokinetic variability or may in fact signal ongoing, silent disease progression. Previous studies have primarily relied on patient-reported outcomes to characterize WoS under NTZ treatment [7,11]. Typically, patients report increased fatigue and cognitive decline shortly before the next NTZ infusion, with symptoms usually improving soon after re-administration [12]. To date, no association has been demonstrated between the WoS phenomenon and clinical disease progression or MRI-based disease activity, including new or enlarging T2 lesions or gadolinium-enhancing lesions [3,11]. A recent study further showed no relationship between WoS and serum neurofilament light chain (NfL) or glial fibrillary acidic protein (GFAP) levels - markers of neuroaxonal and astrocytic damage - supporting the notion that WoS does not reflect ongoing neuroinflammatory disease progression [13]. However, it should be noted that both NfL and GFAP levels remain relatively stable for several months [14] and typically low under NTZ treatment [15]. Moreover, subtle changes in white matter integrity, assessed through diffusion tensor imaging (DTI), may provide a more promising means of detecting MRI changes than conventional measures [16,17].

In addition, digital health technologies (DHTs), such as connected wearables, enable high-frequency collection of real-life data, moving clinical assessment from static to dynamic monitoring [18]. By capturing movement patterns, sleep behavior, and cognition, DHTs are increasingly used in neurology trials to complement conventional measures [19]. This digital phenotyping can provide a comprehensive picture of deficits relevant to patients' daily lives, such as quality of life or motor function [20]. In the context of MS, DHTs hold promise for improving the detection of subtle disease alterations like WoS [21,22].

Against this background, it remains an open question whether different or multimodal approaches - integrating serological, radiological, and digital monitoring tools - could provide deeper insights into the pathophysiology and clinical relevance of WoS or even capture characteristics of it objectively.

## Methods

This prospective, single-center observational study was conducted at the Department of Neurology, University Hospital Düsseldorf, Germany (NCT05701423). A total of 34 patients were enrolled and followed for up to 30 weeks. Key inclusion criteria were age ≥18 years at baseline, a diagnosis of relapsing-remitting MS according to the revised 2017 McDonald criteria [23], and treatment with NTZ for at least 6 months.

Participants received either IV NTZ or SC NTZ at a Q4W (every 4 weeks) or Q6W (every 6 weeks) dosing interval. The choice of SC or IV administration and the respective dosing interval was made prior to study enrollment, based on shared decision-making between patient and treating physician. Neither the route of administration nor the dosing interval was modified during the course of the study.

**Fig. 1:**
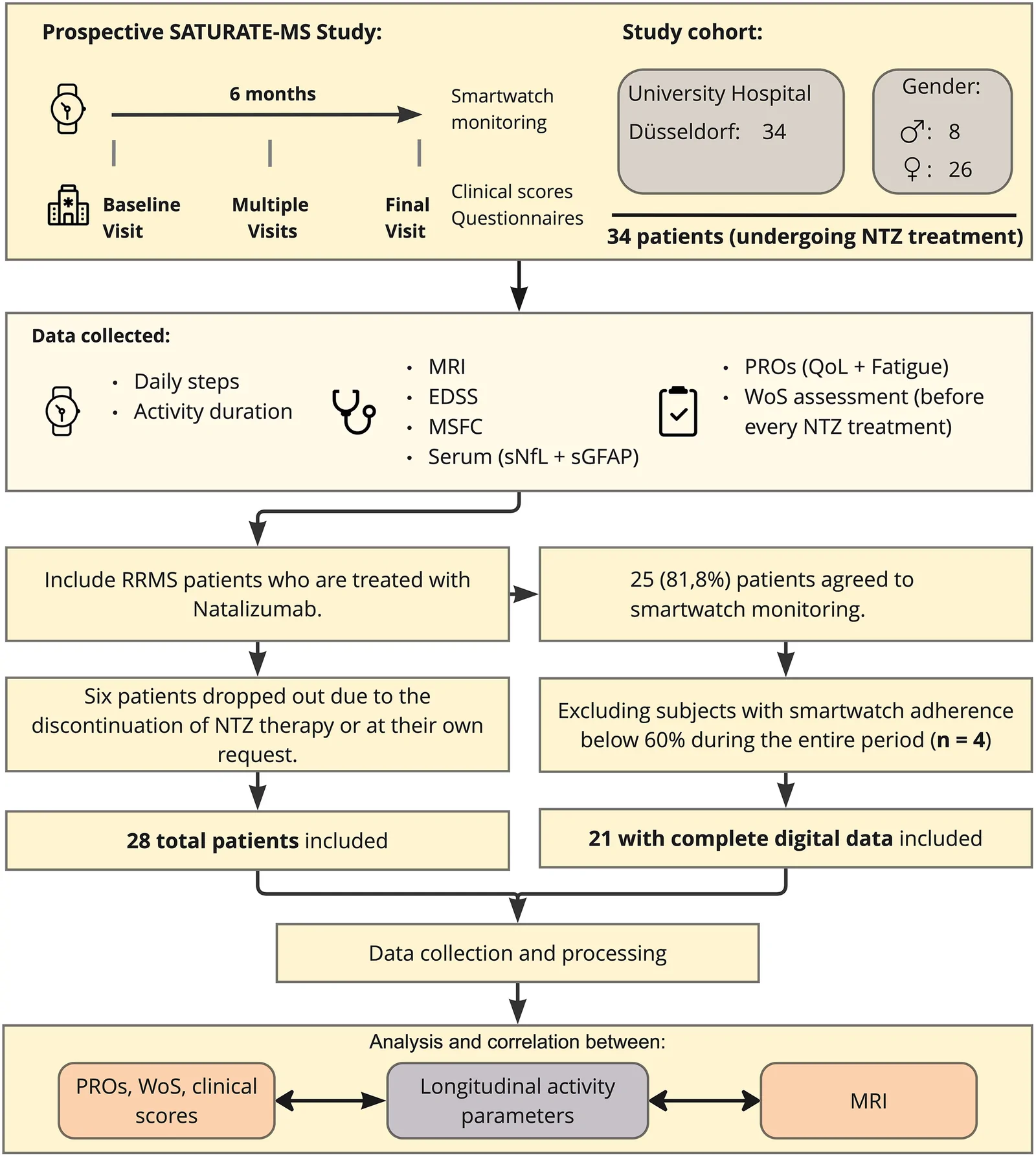
**Study and analysis overview**. This figure illustrates the design and process of the study and its here presented analysis, including patient recruitment, data collection, and analysis. The study followed 34 patients undergoing natalizumab treatment over 6 months, collecting various clinical scores, serological analysis, radiological assessments and smartwatch-derived activity data. Abbreviations: EDSS, Expanded Disability Status Scale; MRI, magnetic resonance imaging; MSFC, Multiple Sclerosis Functional Composite; NTZ, natalizumab; PROs, patient-reported outcomes; sGFAP, serum glial fibrillary acidic protein; sNfL, serum neurofilament light chain; WoS, wearing-off symptoms.

Patients underwent three scheduled study visits at baseline, month 3, and month 6. Clinical assessments were performed at baseline, month 3, and month 6; serum sampling was performed at baseline and month 6 (Fig. 1). Additionally, MRI of the brain was performed at baseline and at the end of the study (month 6). Throughout the study period, patients received NTZ either Q4W or Q6W, depending on their individual dosing regimen. Prior to each administration, participants completed a standardized questionnaire specifically developed to capture the presence of WoS (**Supplemental S1**). An assessment was classified as WoS-positive when the binary general-worsening item was positive. Fatigue and the other symptom-specific items were recorded separately and did not contribute to WoS classification. WoS rate was defined per participant as the number of administrations with any WoS reported on the pre-infusion questionnaire divided by that participant's total number of administrations during follow-up.

Disability status was evaluated using the Expanded Disability Status Scale (EDSS) with changes from baseline analyzed to assess disability progression. Functional performance was further assessed using the Multiple Sclerosis Functional Composite (MSFC), which includes the Timed 25-Foot Walk (T25FW), the 9-Hole Peg Test (9HPT) for both hands, and the Paced Auditory Serial Addition Test (PASAT) to evaluate cognitive processing speed. A composite MSFC Z-score was calculated by averaging the individual Z-scores of the component tests, with higher scores reflecting better functional outcomes [24]. Due to comparably good performance, the cohort was used as its own reference (T25FW: 5.23 ± 2.22 s; 9HPT: 0.0466 ± 0.0098 s^−1^, PASAT: 27.57 ± 9.14).

Fatigue was evaluated using multiple instruments: the Fatigue Scale for Motor and Cognitive Functions (FSMC), a 20-item self-report questionnaire assessing motor and cognitive fatigue (score range: 20–100), the Fatigue Severity Scale (FSS), a 9-item scale where scores ≥36 indicate clinically relevant fatigue, and the Brief Fatigue Inventory (BFI), which measures fatigue severity over the previous 24 h on a 0–10 numerical rating scale.

### MRI measures

MRI was conducted before the administration of NTZ on the administration day. All imaging data were acquired on a Siemens MAGNETOM TrioTim 3 Tesla MRI scanner with syngo MR B17 software and a 12-channel Head Matrix Coil. For anatomical reference, each scanning session included a high-resolution T1-weighted structural scan using a 3D-MPRAGE sequence (TE/TR = 3.26/1900 ms, TI = 900 ms, flip angle = 9°, voxel size = 1.0 mm^3^, FoV: 256 × 256 mm^2^; image matrix: 256 x 256, 192 sagittal slices). Parallel imaging (GRAPPA) with an acceleration factor of 2 was used, yielding a scan duration of 4 min and 26 s.

For the detection of WM lesions, a T2-weighted fluid attenuated (space dark fluid) scan was acquired (TE/TR = 388/5000 ms, TI = 1800 ms, variable flip angle, voxel size = 1.0 mm^3^, FoV = 256 × 256 mm^2^, image matrix = 256 × 256, 192 sagittal slices, GRAPPA acceleration factor = 2, scan duration = 8 min 2 s).

Diffusion-weighted images (DWI) were obtained based on a single-shot, single-refocused echo planar imaging (EPI) pulse sequence (TE/TR = 81/6900 ms, voxel size = 2.4 × 2.4 × 2.4 mm^3^, FoV = 216 × 216 mm^2^, image matrix = 90 × 90, 60 axial slices parallel to the AC-PC line, receiver bandwidth = 1588 Hz/pixel, echo spacing = 0.71 ms, GRAPPA acceleration factor = 2, 24 reference lines, scan duration = 7 min 49 s) using 64 non-collinear diffusion gradients (Siemens MDDW schema) with a b-value of 1000 s/mm^2^ and phase encoding direction from anterior to posterior. To generate appropriate fieldmaps to correct for susceptibility-induced distortions two nondiffusion weighted volumes (b = 0 s/mm^2^) with phase encoding direction from anterior to posterior and one volume with reverse phase encoding direction from posterior to anterior were acquired.

The white matter lesions were counted, and the lesion count was dichotomized into groups of ≥9 and < 9 lesions.

### DWI data preprocessing

The entire diffusion dataset was denoised using the MRtrix3 [25] tool *dwidenoise* [26] and Gibbs ringing was corrected using *mrdegibbs* [27]. After that the diffusion data were processed using the Functional Magnetic Resonance Imaging of the Brain Software Library (FSL, version 6.0.6.4; Oxford, UK). The preprocessing steps included the commands *topup* & *eddy* [28,29] to correct for distortions caused by magnetic field inhomogeneities, eddy currents and motion artifacts respectively. FA (fractional anisotropy), MD (mean diffusivity), AD (axial diffusivity) images were generated using *dtifit* and checked for the presence of artifacts. RD (radial diffusivity) maps were computed as mean of the 2nd and 3rd eigenvalue of the diffusion tensor using *fslmaths*.

### TBSS processing

Voxel-wise statistical analysis was performed using tract-based spatial statistics (TBSS [30]) to evaluate longitudinal differences. The TBSS standard pipeline (https://fsl.fmrib.ox.ac.uk/fsl/docs/#/diffusion/tbss) was applied and all data were projected into the MNI space using the FMRIB58_FA template. Voxelwise statistics on the skeletonized FA/MD/AD/RD data were performed using permutation tests with 10,000 permutations and threshold-free cluster enhancement (TFCE). Longitudinal effects within the group were analyzed by calculating pairwise difference maps for each patient and DTI parameter and one-sample *t*-test. Graphical presentation of results was performed using FSLeyes software (version 1.6.1; Oxford, UK).

The dependence of DTI parameters on clinical parameters was investigated in a GLM, controlling for the age of the subjects at baseline.

### Laboratory measures

Blood samples were collected at two timepoints (Baseline, month 6) from all study participants before NTZ administration via a short catheter from an antecubital vein into Monovettes (Sarstedt, Nümbrecht, Germany). Within 1 h of collection, the samples were transported to the central biobank of the Department of Neurology of the University Hospital Düsseldorf. They were centrifuged at 3000 rpm for 10 min, pipetted, and frozen at −20 °C within 60 min of collection. Every four weeks, the stored samples were transferred to a −80 °C freezer, where they remained until final laboratory NfL and GFAP analysis.

For quantification of both proteins, the samples were sent to the Central Institute for Clinical Chemistry and Laboratory Diagnostics at the Medical Faculty, University Hospital Düsseldorf. Both biomarkers were measured as RUO immunoassays (ECLIA, electrochemiluminescence immunoassay, two-incubation step sandwich assay) according to the manufacturer's instructions using the Roche Cobas 8000 (Roche Elecsys® module). All laboratory technicians performing the analyses were blinded to the clinical data. sGFAP and sNfL Z scores were calculated as described before [[31], [32], [33]]. NTZ (abcam ab282908) concentrations and soluble VCAM-1 (sVCAM-1) levels (R&D Systems DVC00) were measured according to manufacturer's instructions by ELISA as previously reported [34]. Concentrations were reported in μg/mL and ng/mL, respectively.

### Digital measures

After enrolling in the study, a smartwatch (Withings ScanWatch, Withings, Paris) was handed out to patients and set up with their smartphone, which regularly transmitted the data to the GDPR-compliant Withings server, from which the data could be accessed by the researchers. Patients were instructed to wear the smartwatch day and night for the entire duration of the study. The smartwatch and its internal algorithm produced the following longitudinal activity parameters: daily steps as well as the estimated duration of soft (light), moderate, and intense (vigorous) activity (in seconds) according to the Physical Activity Guidelines for Americans [35] and the Compendium of Physical Activities [36]. Furthermore, a more detailed intraday step pattern could be accessed for each user. Sleep and cardiac data were also collected but not part of this analysis, which focused on the step data.

To enable intra-individual comparisons, all aggregated activity parameters were Z-score normalized per subject across the observation period (subtracting the subject mean and dividing by the subject standard deviation). For each metric we computed the median, 10th percentile (Q10), and 90th percentile (Q90) per subject.

### Derived diurnal and dynamic smartwatch activity parameters

From the detailed intraday step data, diurnal activity patterns were analyzed by aggregating steps into four predefined time windows: morning (6:00 a.m.–12:00 p.m.), afternoon (12:00 p.m.–6:00 p.m.), evening (6:00 p.m.–12:00 a.m.), and night (12:00 a.m.–6:00 a.m.). In addition to these absolute segment values, relative measures such as ratios between time windows (e.g., morning-to-afternoon or morning-to-evening step ratios) were calculated to capture intra-daily redistribution of activity. For daytime dependent metrics only patients with more than 500 daily steps were included.

To assess daily performance capacity, we derived “peak steps”, defined as the maximum number of steps recorded in a continuous time window of predefined length and calculated it for the timeframe of 1 h.

To further characterize variability in peak performance, we calculated the relative spread of peak steps, defined as the difference between the 90th and 10th percentiles of daily peak-hour steps (Q90–Q10), divided by the individual's median peak-hour steps. This measure reflects each patient's performance reserve or envelope: a larger relative spread indicates greater day-to-day fluctuation between high and low activity, whereas a smaller spread suggests a narrowed performance range and potentially reduced reserve capacity.

### Smartwatch adherence analysis

For the purpose of activity analysis, adherence was defined as wearing the device for at least 60% of the designated wake hours each day. Wake hours were set from 6:00 a.m. to 11:00 p.m., based on patients’ reported sleep patterns and adjusted for local time zones. Only days meeting these adherence criteria were included in the analysis. Patients who failed to meet adherence on more than 40% of study days or less than a total of 30 days were excluded.

### Testing WoS across baseline parameters

BMI and age were each dichotomized at their sample medians, and WoS-rate was compared between high vs low BMI and older vs younger age groups, as well as across NTZ administration routes (IV vs SC) and treatment frequencies (every 4 weeks vs every 6 weeks), using independent-samples t-tests. A multivariable linear regression model was then fitted with WoS rate as the dependent variable and baseline BMI, age, disease duration, EDSS, route of application and treatment frequency as predictors. The fitted coefficient was reported with HC3 heteroscedasticity-robust standard errors and 95% confidence intervals. Conventional model-based standard errors were additionally reported as a sensitivity analysis to show the dependence of inference on variance estimation.

### Correlation analyses

Associations between smartwatch variables and clinical scores were visualized using multi-panel scatter plots and correlation heatmaps. Missing data were handled by pairwise deletion. Two-sided Spearman rank correlations were used throughout for simplicity and comparability due to the ordinal nature of EDSS and in some cases skewed distributions; scatter plots displayed correlation coefficients with linear trend lines. Heatmaps showed correlations only if determined statistically significant. To account for multiple comparisons in this exploratory setting, p-values were adjusted using false discovery rate control (Benjamini–Hochberg). α was set to 0.05 (two-sided) throughout. Marginal 95% percentile confidence intervals for key correlations were obtained by participant-level bootstrap resampling.

### Segment analyses of treatment cycle

To examine intra-individual changes across treatment cycles, daily measures were aggregated into three predefined segments (beginning, middle, end), excluding infusion days and cycles markedly prolonged for clinical reasons. Participants contributed to a comparison only if data were available for relevant segments. Segment differences in subject-level normalized aggregated step counts and diurnal step metrics were tested using paired Wilcoxon signed-rank tests. In parallel, ordinary least squares models with segments as a categorical predictor were fitted to estimate adjusted segment effects. Inference followed a gatekeeping procedure: a significant omnibus test was required before evaluating prespecified pairwise contrasts (beginning vs end, etc.). Multiplicity across contrasts was more strictly controlled using Holm's step-down procedure, with both raw and adjusted p-values reported. For these analyses, only cycles with wearable data on more than 75% of days and with regular application intervals were included. For an exploratory comparison of WoS-positive and WoS-negative cycles, end-minus-beginning changes were calculated for each eligible cycle. Among participants contributing both cycle types, changes were averaged within participant and WoS status and compared using participant-level bootstrap confidence intervals and exact sign-flip tests, with Holm correction across total, morning, afternoon, and evening step outcomes.

### Software

Analyses were performed using Python 3.12.9 (Python Software Foundation, Delaware, USA) with the SciPy package version 1.15.3, Pandas package version 2.2.3, Statsmodels package version 0.14.4, Scikit-posthocs package version 0.11.4 and the NumPy package version 2.2.5. The results were visualized using matplotlib version 3.10.3. A web application to synchronize, organize and visualize data was set up based on the dash framework version 2.6.1.

## Results

From 34 patients included, a total of 28 patients completed the study and had sufficient datasets for analysis. Baseline demographics are summarized in Table 1. Adherence to smartwatch use was very high throughout the study period, with 21 of 25 (84%) patients that opted into wearable-monitoring meeting the adherence target (Supplemental Fig. 1). Clinical, MRI, and serological analyses included all 28 participants with available baseline and month-6 EDSS, while wearable-based analyses comprised 21 participants with sufficient data.

**Table 1:** Cohort statistics.

		Missing/n	Overall
**Finished study and complete dataset – included in analysis, n (%)**		0/34	28 (82.4)
**Sex, n (%)**	**Female**	0/22	22 (78.6)
	**Male**	0/6	6 (21.4)
**Age at inclusion [y], mean (SD)**		0/28	37.6 (12.7)
**Age at diagnosis [y], mean (SD)**		0/28	25.2 (6.8)
**Disease duration [y], median [Q1,Q3]**		0/28	10.0 [2.75,21.5]
**Baseline EDSS, median [Q1,Q3]**		0/28	2 [1.5,2.5]
**Positive anti-JC virus serology, n (%)**		0/28	9 (32.1)
**Baseline natalizumab concentration (μg/ml), median [Q1,Q3]**		6/28	12.57 [6.51, 27.10]
**EoS natalizumab concentration (μg/ml), median [Q1,Q3]**		6/28	18.11 [11.55, 25.53]
**Baseline sVCAM-1 concentration (ng/ml), median [Q1,Q3]**		6/28	106.77 [84.55, 138.99]
**EoS sVCAM-1 concentration (ng/ml), median [Q1,Q3]**		6/28	83.09 [77.81, 117.84]
**Duration of natalizumab treatment [m], median [Q1-Q3]**		0/28	77 [24,158]
**Natalizumab interval, n (%)**	**4w**	0/28	19 (67.9)
	**6w**		9 (32.1)
**Natalizumab application, n (%)**	**SC**	0/28	12 (42.9)
	**IV**		16 (57.1)
**Number of previous DMTs, median [Q1,Q3]**		0/28	1 [1–2]
**BMI (kg/m^2^), median [Q1,Q3]**		0/28	23.0 [21.6,27.4]
**Visits with reported WoS, n (%)**		0/177	63 (35.6)
**Patients agreed to optional wearable, n (%)**		0/28	25 (89.3)
**Hourly adherence rate digital device (%), median [Q1, Q3]**		0/25	98.8 [82.6, 99.3]
**Patients meeting wearable adherence target, n (% of all wearable patients)**		0/25	21 (84.0)

Baseline characteristics of the study cohort. Data are shown as absolute numbers and percentages (n [%]), means with standard deviation (SD), or medians with interquartile range (Q1–Q3), as appropriate.

Abbreviations: BMI = Body Mass Index; DMT = Disease-Modifying Therapy; EoS = End of study, EDSS = Expanded Disability Status Scale; IV = intravenous; m = months; n = number; Q1 = first quartile; Q3 = third quartile; SC = subcutaneous; SD = standard deviation; sVCAM-1 = soluble vascular cell adhesion molecule-1; WoS = Wearing off Symptoms; y = years.

All patients reported having experienced WoS in the past under NTZ, and 19 (68%) patients reported at least one WoS episode during the study period (Table 1). Common WoS reported by patients were a worsening of fatigue (reported by 68% of all patients, n = 19), cognition (61%, n = 17), pain (57%, n = 16), sleep (54%, n = 15), and spasticity (39%, n = 11) or walking (36%, n = 10) symptoms. WoS were reported at 63 (36%) of 177 observed pre-administration questionnaires. Univariate tests showed no differences by age (t (24.44) = 0.43, p = 0.668), BMI (t (23.57) = 0.86, p = 0.397) or treatment frequency (t (12.93) = –0.58, p = 0.574) regarding the rate of WoS, but SC patients had a lower WoS rate than IV patients (0.188 ± 0.193 vs. 0.489 ± 0.398; t (22.81) = 2.64, p = 0.015). In the adjusted model, the SC-versus-IV coefficient was −0.296 (R^2^ = 0.361). The association was not statistically significant using HC3-robust standard errors (95% CI, −0.649 to 0.056; p = 0.100), whereas conventional model-based standard errors yielded a narrower interval (95% CI, −0.575 to −0.018; p = 0.038). Inference regarding route was therefore sensitive to variance estimation.

Over the course of the study, no clinical deterioration was observed based on EDSS or MSFC (sub-) scores (Table 2). Only one patient showed disease activity (new MRI lesion, but no report of clinical relapse).

**Table 2:** Comparing clinical and serological markers between baseline and end-of-study.

Measure	N	Baseline	EoS	Mean difference	Test	Statistic	P-value	P-value (holm corrected)	Effect size (d)	Significance	Significance (holm corrected)
**EDSS, median [Q1-Q3]**	28	2.00 (1.50–2.62)	2.00 (1.50–2.5)	0.09	Wilcoxon signed-rank test	30.000	0.244	0.977	0.22	ns	ns
**MSFC Z-score, mean (SD)**	28	0.09 ± 0.68	0.32 ± 0.79	0.23	Paired *t*-test	−2.816	0.009	0.063	0.53	∗∗	ns
**PASAT Z-score, mean (SD)**	28	0.06 ± 0.99	0.80 ± 1.48	0.74	Paired *t*-test	−3.629	0.001	0.009	0.69	∗∗	∗∗
**9-HPT Z-score, mean (SD)**	28	0.04 ± 0.96	0.20 ± 1.03	0.17	Paired *t*-test	−1.894	0.069	0.392	0.36	ns	ns
**T25FW Z-score, median [Q1-Q3]**	28	0.22 (0.11–0.56)	0.11 (−0.12–0.39)	−0.22	Wilcoxon signed-rank test	32.500	0.065	0.392	−0.35	ns	ns
**GFAP Z-score, mean (SD)**	27	0.16 ± 1.35	0.34 ± 1.12	0.18	Paired *t*-test	−1.093	0.284	0.977	0.21	ns	ns
**sNfL Z-score, mean (SD)**	27	−0.18 ± 0.87	−0.37 ± 1.08	−0.19	Paired *t*-test	0.973	0.339	0.977	−0.19	ns	ns
**FSMC total score, mean (SD)**	28	62.39 ± 19.31	62.96 ± 21.33	0.57	Paired *t*-test	−0.337	0.739	0.977	0.06	ns	ns

Values are presented as mean ± standard deviation (SD) for normally distributed differences, or median (interquartile range, IQR) for non-normally distributed differences.

Abbreviations: 9-HPT = 9-Hole Peg Test; EDSS = Expanded Disability Status Scale; EoS = End of Study; FSMC = Fatigue Scale for Motor and Cognitive Functions; GFAP = Glial Fibrillary Acidic Protein; MSFC = Multiple Sclerosis Functional Composite; PASAT = Paced Auditory Serial Addition Test; sNfL = serum neurofilament light chain; T25FW = Timed 25-Foot Walk.

Statistical significance: ∗∗∗p < 0.001, ∗∗p < 0.01, ∗p < 0.05, ns = not significant. Both uncorrected and Holm-corrected p-values are provided.

**Fig. 2:**
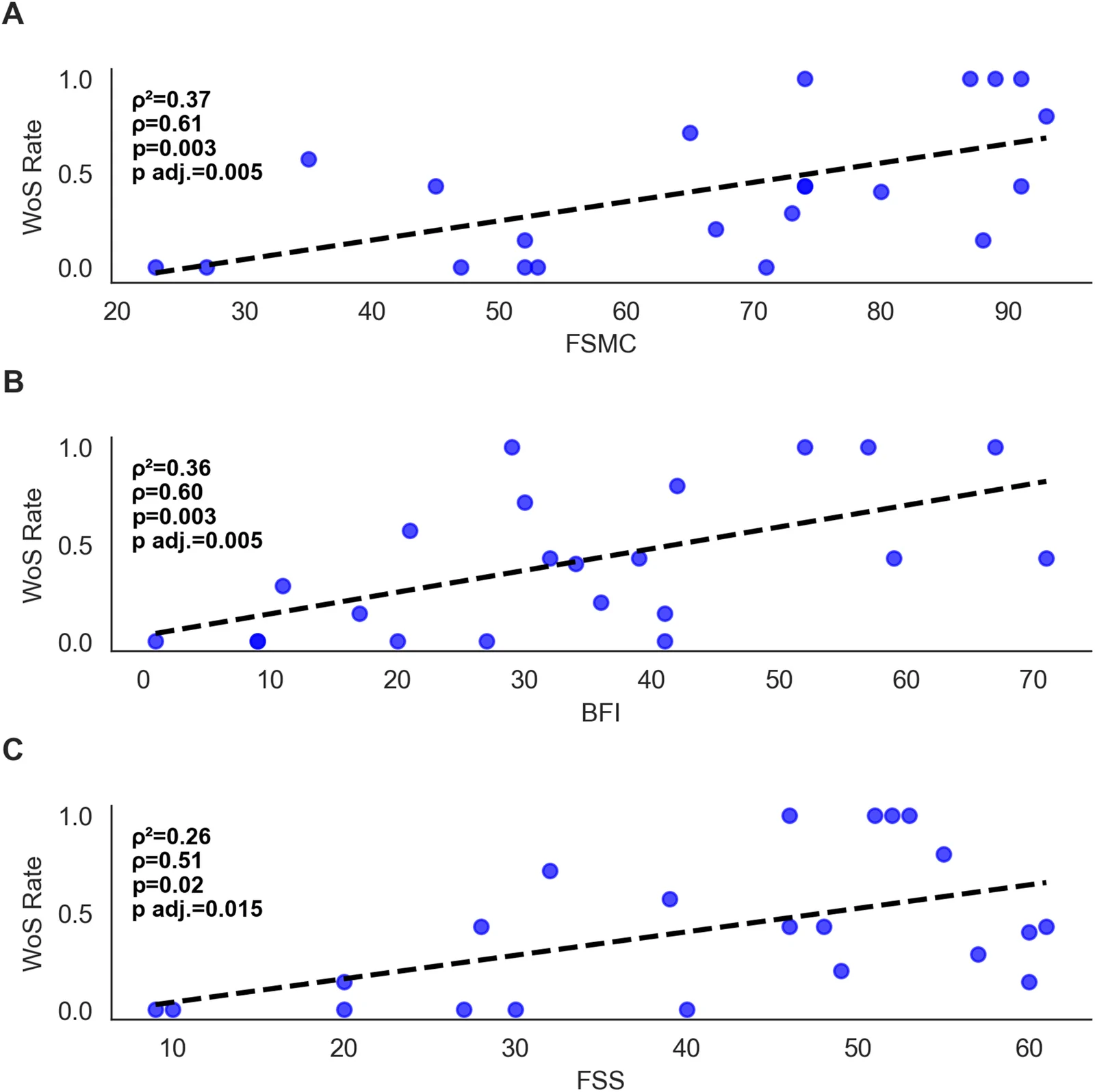
**Association Between Wearing-Off Symptoms Rate and Fatigue Scores** Scatter plots with linear regression lines show the relationship between the rate of reported wearing-off symptoms (WoS rate) and fatigue severity as measured by (A) the FSMC, (B) the BFI, and (C) the FSS. Each point represents an individual patient. Spearman correlation coefficients (ρ) and their squared values (ρ^2^), as well as p-values are provided for each association. **Abbreviations:** BFI, Brief Fatigue Inventory; FSS, Fatigue Severity Scale; FSMC, Fatigue Scale for Motor and Cognitive Functions; WoS, Wearing-off Symptoms.

***Association Between WoS Rate and Fatigue.*** The frequency of WoS reported during the study showed a clear and consistent association with multiple fatigue measures. WoS rate correlated with FSMC (R = 0.608; 95% CI, 0.237–0.837; p = 0.0027; BH-adjusted p = 0.0046), BFI (R = 0.601; 95% CI, 0.225–0.825; p = 0.0031; adjusted p = 0.0046), and FSS (R = 0.510; 95% CI, 0.085–0.760; p = 0.0153; adjusted p = 0.0153) scores. (Fig. 2). In contrast, WoS rate was not correlated with EDSS (R = 0.278; 95% CI, −0.199 to 0.701; p = 0.210) or MSFC (R = 0.178; 95% CI, −0.282 to 0.608; p = 0.427).

### WoS and its association with diffusion MRI analyses

Subsequently, we examined whether WoS occurrence and fatigue severity could be reflected in radiological changes. MRI assessments across the cohort showed no dynamic changes over time with respect to lesion count groups (Suppl. Table 1).

TBSS analyses revealed no significant changes in MD, RD, AD, or FA within the white matter tracts of the brain across the observation period in the overall cohort. We then investigated whether the WoS rate and fatigue scores (BFI, FSMC, FSS) correlated with diffusion metrics at baseline and at month 6. Again, no meaningful correlations were found. Finally, we examined whether higher rates of WoS and elevated fatigue scores were associated with longitudinal changes in diffusion parameters between baseline and month 6. No significant associations were identified.

### WoS and its association with serum biomarkers

For sNfL and sGFAP levels, we detected only minor variations within the entire cohort, which did not correlate with WoS frequency or fatigue severity at baseline and month 6. Z-score levels were very low. Only one patient showed a relevant increase with the above-mentioned corresponding radiological activity. With respect to NTZ concentrations and sVCAM-1 levels, we observed no relevant differences between application variants or dosing intervals, and no association with WoS or fatigue severity.

### Wearable-derived activity patterns mirror WoS dynamics

**Fig. 3:**
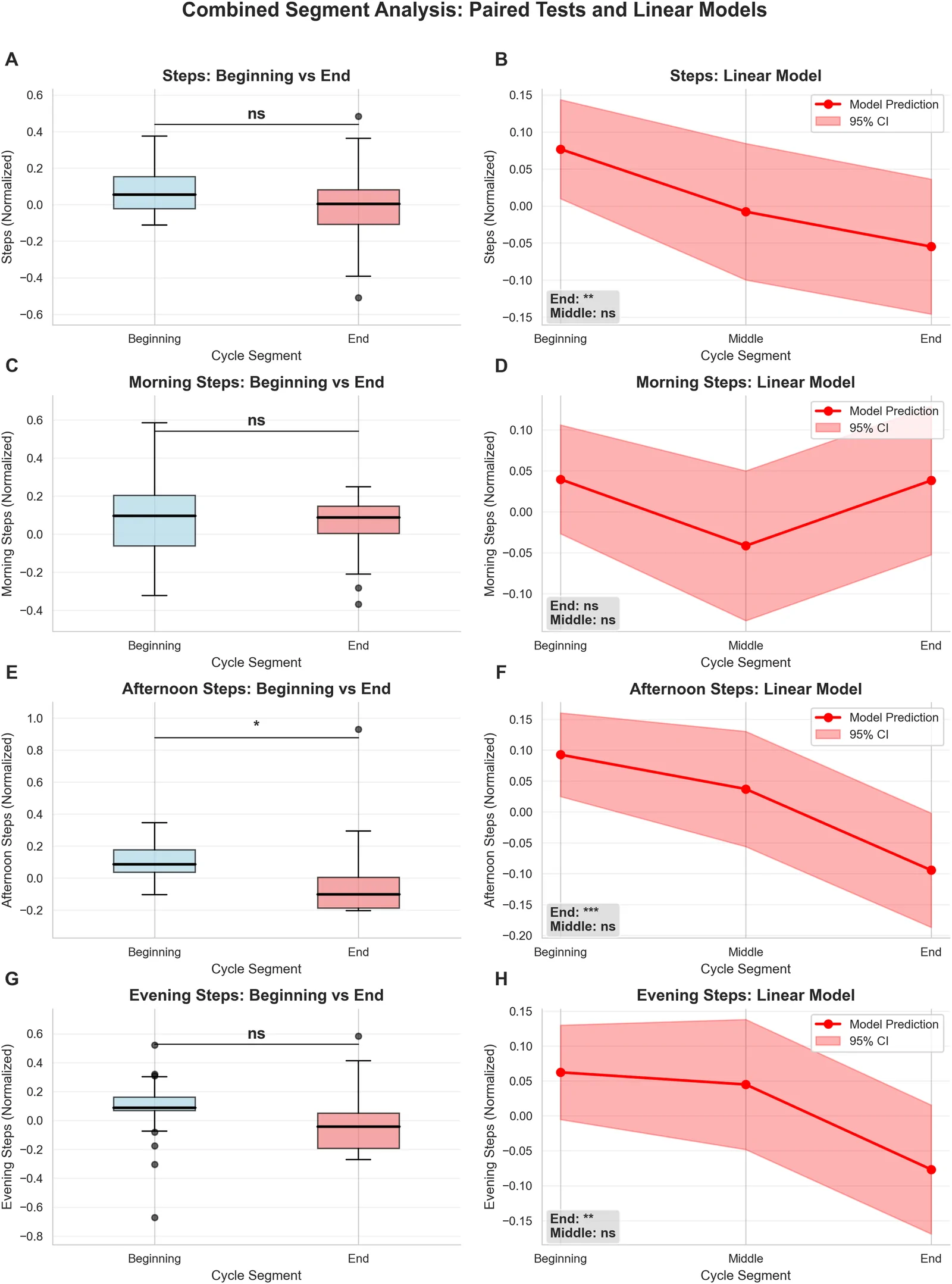
**Wearable-Derived Activity Patterns Across Infusion Cycle** Boxplots (A, C, E, G) and linear-model predictions (B, D, F, H) show participant-normalized step counts across cycle segments (beginning, middle, end) for total daily steps, and morning, afternoon, and evening steps. Values were centered to the subject's mean and scaled by the subject's standard deviation. Boxplots display medians, IQRs, and outliers as points; paired beginning vs end tests are Wilcoxon tests with Holm correction across the four metrics. Afternoon steps were lower at end (beginning 0.094 ± 0.121 vs End −0.036 ± 0.249; Wilcoxon p = 0.0038, Holm-corrected p = 0.015; n = 21). Other paired comparisons were not significant after correction (total p = 0.24; morning p = 0.71; evening p = 0.16). Linear-model panels plot predicted means with 95% CIs. End vs beginning contrasts are Holm-corrected within panel and gated by the omnibus effect: Differences between initial and last segment were significant for total steps (Holm-adjusted p = 0.0094). There were also significant differences for afternoon steps (adjusted p = 0.0002) and evening steps (adjusted p = 0.0063). Morning steps were not significant after omnibus gating (omnibus p = 0.13). Middle vs beginning contrasts were not significant for any measure.

We next investigated whether wearable-derived data could reflect WoS frequency. Due to the frequent reporting of motor symptoms as WoS, we first explored whether smartwatch-based activity monitoring could capture subtle motor changes not evident in clinical assessment that might be associated with the NTZ treatment cycle. A total of 88 NTZ treatment cycles could be included based on regular infusion intervals and completeness of data, comprising 34 WoS-positive and 54 WoS-negative cycles. Patient-level normalized mean daily activity declined slightly and selectively toward the end of the cycle. In paired comparisons of the first vs last cycle segment, only afternoon steps were lower at the end (beginning mean z-score 0.094, SD 0.121 vs end −0.036, SD 0.249; mean difference −0.130; Wilcoxon p = 0.0038, Holm-corrected p = 0.015; n = 21). Differences for total steps (p = 0.12), morning steps (p = 0.71), and evening steps (p = 0.055) were not significant. In a linear model with cycle segment as predictor and disease duration and EDSS as covariates, the end segment showed lower activity than the beginning for total steps (p = 0.0094), afternoon steps (p = 0.0002), and evening steps (p = 0.0063), while morning steps showed no effect (omnibus p = 0.13; no pairwise comparisons). The decline was most pronounced for afternoon steps. Changes in the step patterns were not associated with disease duration or EDSS. There was no difference between middle and beginning segments for any step measure (see Fig. 3, see Suppl. Tables 2–5 for model information and coefficients).

In an exploratory within-person analysis of seven participants contributing both cycle types, the WoS-positive-minus-WoS-negative difference in total-step change was −0.148 SD units (95% CI, −0.458 to 0.167; Holm-adjusted p = 1.000), with no detectable differences for any diurnal outcome (all 95% CIs included zero; Holm-adjusted p ≥ 0.563).

### Digital measures reveal WoS and its association with fatigue

While our data could confirm general moderate-to-high correlations of daily step counts (and steps across different times of day) to clinical scores such as MSFC or EDSS (Supplemental Fig. 2), there were no significant correlations to WoS frequency or fatigue scores after multiple testing correction (Supplemental Fig. 3). We then explored whether more dynamic measurements of performance patterns captured by the wearables could serve as indicators of WoS and fatigue.

**Fig. 4:**
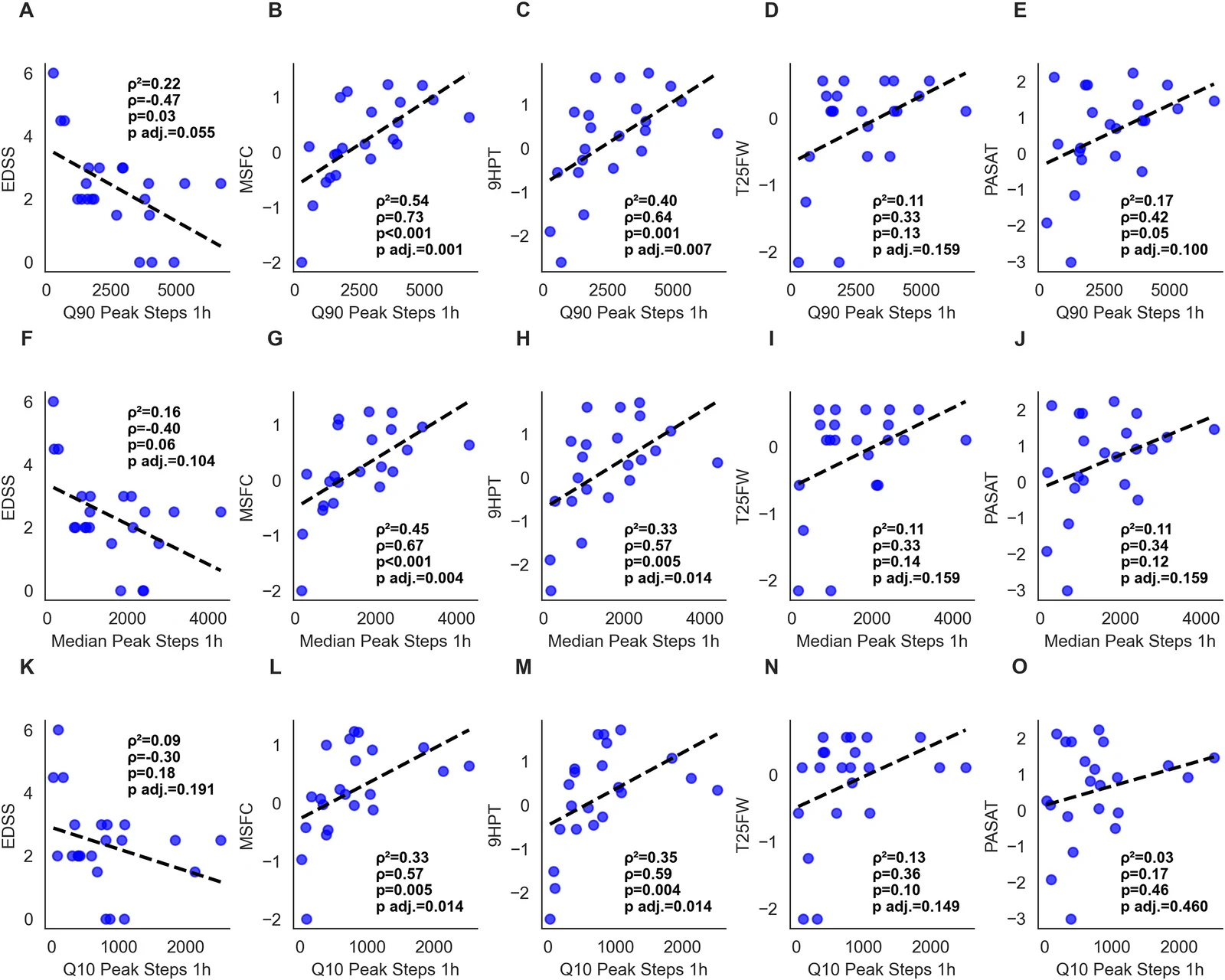
**Associations Between Physical Activity Metrics and Clinical Disability Measures** Scatter plots with linear regression lines show associations between different physical activity parameters and neurological disability measures. The top row (A–E) uses the 90th percentile of hourly peak step counts (Q90), the middle row (F–J) uses the median of daily peak step counts (Median Peak Steps 1 h), and the bottom row (K–O) uses the lowest 10% of daily peak step counts (Q10). Each column corresponds to a clinical outcome: EDSS, MSFC, 9HPT, T25FW, and PASAT. Each dot represents one patient. Spearman correlation coefficients (ρ) and its squared value (ρ^2^) as well as p-values are provided in each subplot. False discovery rate was controlled using Benjamini-Hochberg method. Abbreviations: 9HPT, 9-Hole Peg Test; EDSS, Expanded Disability Status Scale; MSFC, Multiple Sclerosis Functional Composite; PASAT, Paced Auditory Serial Addition Test; Q10, lowest 10% of daily step counts; Q90, 90th percentile of hourly peak step counts; T25FW, Timed 25-Foot Walk.

We summarized each person's best 60-min window per day - their peak-hour steps - and looked at how these peak-step measurements compare to other clinical indicators. Firstly, we wanted to compare peak steps as well as the 10th and 90th percentiles against established correlations to clinical scores and they showed again moderate-to-high correlation to some clinical measures, particularly MSFC, with the 90th percentile performing best (Fig. 4).

Given the correlation of our dynamic measures with clinical variables, we then developed additional dynamic measures more representative of clinical fatigue. For this we chose three approaches.

- Relative peak steps spread (Q90 − Q10), a potential reserve-capacity measure where smaller values reflect a narrowed performance envelope and higher fatigue
- Normalized peak steps, expressed relative to each person's distribution, where for example smaller values of the 10th percentile indicate a smaller spread and potentially higher fatigue
- and, after our initial results, diurnal ratios of steps during different times of day (morning:afternoon and morning:night), where higher ratios indicate comparatively calmer afternoons or nights, consistent with a need to rest later in the day.

**Fig. 5:**
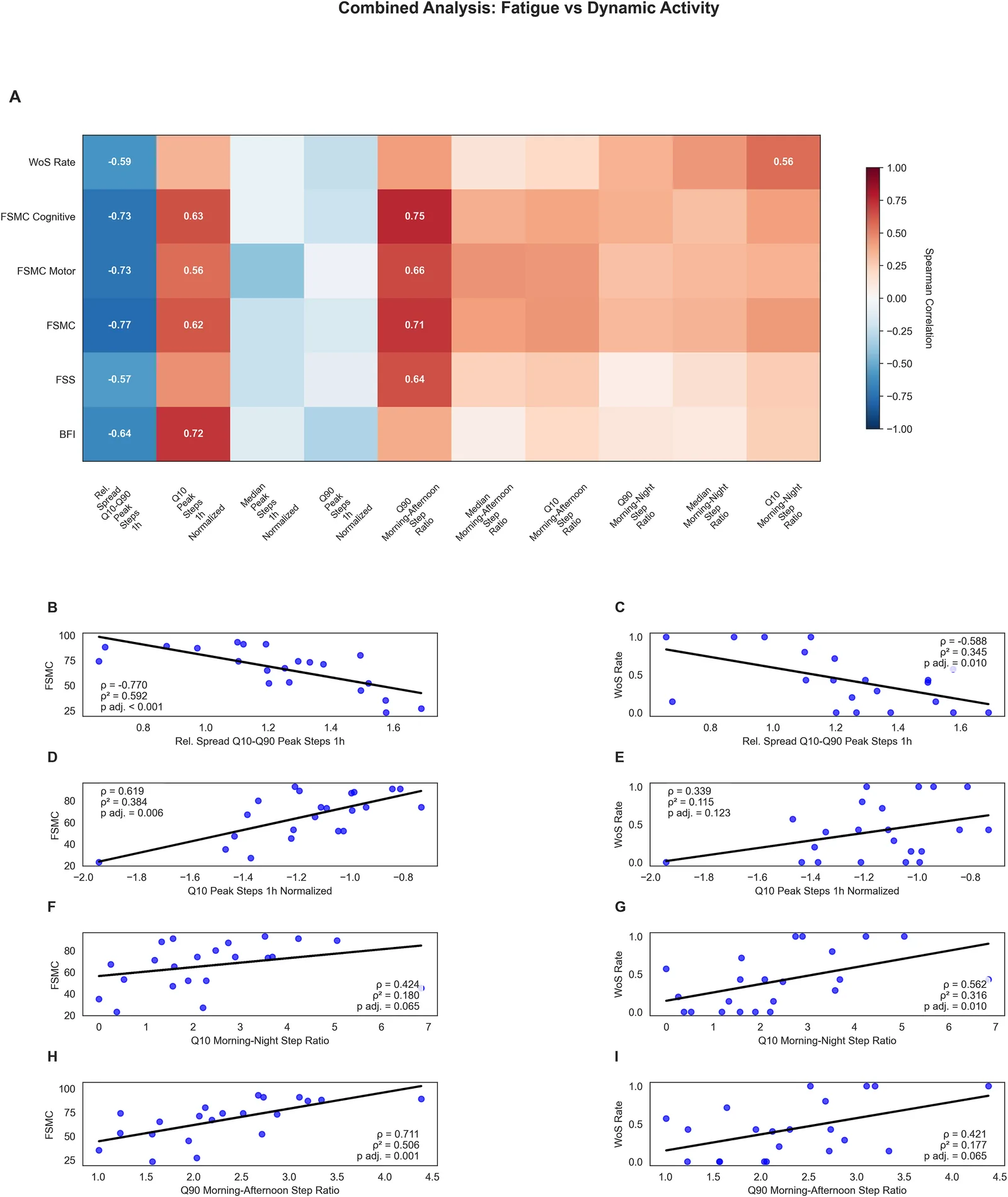
**Combined Analysis of Fatigue and Wearing-Off Symptoms vs. Dynamic Step Count Features** (A) Heatmap showing Spearman correlation coefficients between fatigue measures, WoS rate, and various dynamic step count features, including temporal distributions and activity ratios (e.g. morning–night). Strong negative associations were observed between fatigue scales (FSMC, FSS, BFI) and measures of activity fragmentation and morning activity decline. (B–I) Scatter plots illustrate selected exploratory associations between dynamic physical activity features and fatigue scores (FSMC) or WoS Rate, including relative spread of activity (Relative Q10–Q90 spread denotes the percentile range and was calculated as (Q90−Q10)/median peak-hour steps), normalized peak steps, and ratios between different times of day (e.g. Q10 Morning–Night Step Ratio). Linear regression lines with Spearman correlation coefficients (ρ) and its squared value (ρ^2^) and p-values are shown for each model. **Abbreviations:** BFI, Brief Fatigue Inventory; FSMC, Fatigue Scale for Motor and Cognitive Functions; FSS, Fatigue Severity Scale; Q10, lowest 10% of step counts; Q90, 90th percentile of peak step counts; WoS, Wearing-Off Symptoms.

We tested different setups and quantiles of these latter two and while not all derived variables correlated strongly with WoS and fatigue scales, three measures stood out: relative peak step spread showed consistent associations across all WoS and fatigue scores, whereas the 10th percentile of normalized peak steps and the 90th percentile of morning-afternoon step ratio were particularly linked to fatigue scales such as FSMC (Fig. 5). These further did not correlate with EDSS or MSFC (Supplemental Fig. 4).

## Discussion

WoS are frequently encountered in clinical practice and often raise concerns about subclinical disease activity. In this prospective study, a multimodal readout spanning clinical exams, serum biomarkers, diffusion MRI, and continuous wearable monitoring did not reveal evidence of ongoing inflammatory or neurodegenerative progression in patients reporting WoS. Instead, the digital signal associated with WoS mapped more closely to fatigue-related reductions in motor activity. As serum biomarkers and MRI were assessed only at baseline and month 6, these analyses do not address short-term biological fluctuations within individual NTZ treatment cycles. WoS appeared less frequent with SC than IV administration in adjusted models. Dosing intervals however did not affect abundance of WoS.

Given the observational design, small sample size, and potential confounding by indication, these findings require confirmation in randomized or pharmacokinetically phenotyped cohorts. They nevertheless suggest that simple schedule changes are unlikely to fully explain WoS at the group level.

Symptoms are often reported to increase toward the end of the dosing interval and to remit after infusion [7,11,12]. We likewise observed frequent WoS, but found no association with dosing interval, despite assumptions that decreasing receptor saturation or BMI-related variability in SC absorption might drive symptoms [10,37]. Although EID is robustly associated with a lower PML risk and recent data suggest that immune cell trafficking into the CSF and CNS may resume at lower NTZ concentrations [34,38], NTZ serum concentrations and sVCAM-1 levels were not associated with WoS or fatigue in our cohort, although we believe the study was too small to reliably characterize pharmacodynamic dependant effects. Of note, this would also be in contrast to the population-level separation demonstrated in the NOVA study [3]. Our diffusion MRI analyses further support the finding of little tissue damage by showing no detectable microstructural white matter changes associated with WoS, even though such alterations often precede visible lesions on conventional MRI and may reflect diffuse axonal damage not captured by standard imaging techniques [16,17,[39], [40], [41]]. In addition, consistent with a recently published study [13], we found no association between WoS and serum biomarkers such as NfL and GFAP measured at baseline or month 6. These findings strengthen the notion that WoS may not indicate true disease progression but rather reflect subjective symptom perception or threshold effects that are unrelated to inflammatory or neurodegenerative activity. It is, however, important to note that the sampling schedule was not designed to detect short-term within cycle changes and findings therefore remain inconclusive regarding a transient biomarker correlate of WoS.

### Digital signatures and added value

Continuous smartwatch monitoring captured a subtle, temporally reproducible decline in activity toward the end of the infusion cycle, most pronounced in the afternoon. These patterns were not evident in median daily step counts but emerged when trajectories were aligned to cycle timing. This contrasts with a retrospective analysis under anti-CD20 therapy that relied on monthly averages and was not time-locked to infusion cycles [42]. Furthermore, dynamic features derived from intraday activity metrics - e.g., relative peak-hour spread and morning-to-afternoon ratios - tracked fatigue burden better than aggregate daily totals. While prior work connected EDSS to daily steps [21,43], our results extend this by showing that dynamic and diurnal metrics are more informative for fatigue. As fatigue is inherently difficult to measure objectively, these digital approaches could help objectify typical fatigue patterns at comparably low cost using wearable devices in the future.

In aggregate, this leads us to two clinical implications. First, in clinically stable patients with reassuring radiological, serological, and examination findings, WoS should not automatically be interpreted as breakthrough disease. However, our exploratory data do not exclude such a relationship in other clinical contexts. Second, clinicians should assess and manage fatigue proactively, as higher fatigue scores were consistently associated with higher WoS rates. Passive, high-adherence wearable monitoring is well suited in this context and imposes low burden compared with task-based apps or frequent PROs, with adherence comparable to or better than interactive digital platforms [44,45]. While the association between fatigue severity and WoS frequency was robust, directionality cannot be inferred from our current results. Interventional studies that target fatigue should test whether reducing fatigue also reduces WoS.

## Limitations

A key limitation of this study is the relatively small sample size and single-center design, which may affect generalizability. Given the exploratory design and small sample size, the analyses were not powered to exclude clinically relevant associations, and both false-negative and false-positive findings remain possible. Moreover, biomarker and MRI assessments at only two time points could not capture within-cycle biology, while the low level of breakthrough activity limits extrapolation to less stable or recently escalated populations. However, this reflects the complexity and resource intensity of the comprehensive, high-frequency multimodal monitoring protocol including clinical, serological, MRI-based, and continuous digital assessments. Residual confounding is possible, including baseline fatigue, sleep, mood, pain, work patterns, and medication timing. Future larger-scale studies are needed to validate these findings and further explore the mechanistic underpinnings of WoS in diverse MS populations under different therapies. Further, the observational design might have introduced selection bias such as related to treatment choice and prior wearing-off symptoms that authors are unaware of. Lastly, although fatigue did not contribute mathematically to WoS classification, both constructs were patient-reported and overlap clinically. Shared symptom perception and common-method effects may therefore contribute to their association, and directionality cannot be inferred.

To sum up, WoS are a common and clinically relevant phenomenon in PwMS receiving NTZ, often raising concerns about subclinical disease activity. In our cohort, WoS were not associated with radiologic, serologic, or clinical markers of disease progression, but were instead closely related to decreased motor activity and severity of fatigue. Continuous digital monitoring using wearables enabled the objective detection of these subtle symptom dynamics and revealed fluctuations in motor performance within an interval that were undetectable with standard examinations. These findings underscore the value of high-resolution digital tools for distinguishing between subjective symptom perception and actual disease activity and support their role in individualized fatigue-focused treatment strategies, potentially as candidate digital biomarkers.

## Ethics approval and consent to participate

This study was approved by the Ethics Committee of the Medical Faculty Düsseldorf (No.: 2022–2035). The informed consent form was reviewed by the ethics committee for approval of the study and all trial activities were in accordance with the Declaration of Helsinki.

## Availability of data and materials

The data that support the findings of this study are available on request from the corresponding author. The data are not publicly available due to privacy or ethical restrictions. Requests will be processed within six weeks and are subject to the data protection guidelines of University Hospital Düsseldorf.

## Authors' contributions

LM contributed to study conception, study design, formal analysis, data interpretation, visualization, and drafting of the manuscript. ET contributed to data acquisition, data curation, and critical revision of the manuscript. JvP contributed to data acquisition, data curation, and critical revision of the manuscript. RH contributed to data acquisition, data curation, and critical revision of the manuscript. MV contributed to methodological aspects, data interpretation, and critical revision of the manuscript. PK contributed to data acquisition, data curation, and critical revision of the manuscript. JV contributed to data acquisition, data curation, and critical revision of the manuscript. MS contributed to data acquisition, data curation, and critical revision of the manuscript. PB contributed to biomarker methodology, data interpretation, and critical revision of the manuscript. JK contributed to biomarker methodology, data interpretation, and critical revision of the manuscript. JK contributed to methodological aspects, data interpretation, and critical revision of the manuscript. MD contributed to methodological aspects, data interpretation, and critical revision of the manuscript. NS contributed to methodological aspects, data interpretation, and critical revision of the manuscript. SP contributed to study design, data interpretation, and critical revision of the manuscript. SGM contributed to study design, study supervision, data interpretation, and critical revision of the manuscript. MP contributed to study conception, study design, study supervision, data interpretation, and drafting of the manuscript.

## Funding

This study was supported by 10.13039/100005614Biogen through a Sponsored Research Agreement.

## Declaration of interest

LM has received honoraria for lecturing, consulting, and travel expenses for attending meetings from Biogen, Merck, Sanofi, argenX, Roche, Neuraxpharm, Alexion, and Novartis, all outside the scope of this work. His research is funded by the German Multiple Sclerosis Foundation (DMSG) and the Deutsche Forschungsgemeinschaft (DFG, German Research Foundation) – 493,659,010, Roche, Biogen and Neuraxpharm.

ET reports no conflicts of interest.

JvP reports no conflicts of interest.

RH reports no conflicts of interest.

MV reports no conflicts of interest.

PK declares that there are no conflicts of interest in connection with this study, she has received honoraria for lectures from Merck and travel support for attending meetings from the German Neurological Society (DGN), Roche, Sanofi, Merck, Neuraxpharm and Viatris.

JV reports no conflict of interests. He has received travel support for attending meetings from argenX.

MS reports no conflicts of interest related to this study. She reports honoraria for lecturing, consulting and travel expenses for attending meetings from Biogen, Hexal, Janssen Cilag, Juvise, Merck Healthcare Germany, Neuraxpharm, Novartis, Roche, Sanofi, Teva und Viatris. Her research is funded by the German Multiple Sclerosis Foundation (DMSG), Novartis, Neuraxpharm, Roche and Viatris.

PB reports no conflicts of interest.

JK received speaker fees, research support, travel support, and/or served on advisory boards by Swiss MS Society, Swiss National Research Foundation (320,030_212,534/1), University of Basel, Progressive MS Alliance, Alnylam, Bayer, Biogen, Bristol Myers Squibb, Celgene, Immunic, Merck, Neurogenesis, Novartis, Octave Bioscience, Quanterix, Roche, Sanofi, Stata DX.

JK reports no conflict of interest related to this study.

MD reports no conflicts of interest.

NS reports no conflicts of interest.

SP received honoraria for lecturing and consulting from argenx, Sanofi-Avents, Merck Healthcare, Biogen, Novartis Pharma, Roche, Alexion and Hexal, travel reimbursements from Sanofi-Aventis, Merck Healthcare, Novartis Pharma, Roche, and Biogen, and research funding from Merck Healthcare, Novartis Pharma and Biogen.

SGM has received honoraria for lecturing and travel expenses for attending meetings from Almirall, Amicus Therapeutics Germany, ArgenX, Bayer Health Care, Biogen, Celgene, Diamed, Genzyme, MedDay Pharmaceuticals, Merck Serono, Novartis, Neuraxpharm, Novo Nordisk, ONO Pharma, Roche, Sanofi-Aventis, Chugai Pharma, QuintilesIMS, and Teva. His research is funded by the German Ministry for Education and Research (BMBF), Bundesinstitut für Risikobewertung (BfR), Deutsche Forschungsgemeinschaft (DFG), Else Kröner Fresenius Foundation, Gemeinsamer Bundesausschuss (G-BA), German Academic Exchange Service, Hertie Foundation, Interdisciplinary Center for Clinical Studies (IZKF) Muenster, German Foundation Neurology, and by Alexion, Almirall, Amicus Therapeutics Germany, Biogen, Diamed, Fresenius Medical Care, Genzyme, HERZ Burgdorf, Merck Serono, Novartis, ONO Pharma, Roche, and Teva, all outside the scope of this study.

MP has received honoraria for lecturing and travel expenses for attending meetings from Alexion, ArgenX, Bayer Health Care, Biogen, Hexal, Merck Serono, Neuraxpharm, Novartis, Roche, Sanofi-Aventis, Takeda, and Teva. His research is funded by ArgenX, Biogen, Hexal, and Novartis, all outside the scope of this study.
