# Supplementary material for: Digital signatures of fatigue and wearing-off symptoms in patients with multiple sclerosis under natalizumab
Source: Neurotherapeutics. 2026 Sep 18;23(6):e01075. doi: 10.1016/j.neurot.2026.e01075 (PMC13602057; doi:10.1016/j.neurot.2026.e01075)
Supplement: Supplementary file 1 [file mmc1.docx]

[**Supplementary Figure 1: Smartwatch adherence among study participants**	2](#_Toc207904696)

[**Supplementary Figure 2: Selected Smartwatch Variables vs. Clinical Scores**	3](#_Toc207904697)

[**Supplementary Figure 3: Selected Smartwatch Variables vs. Fatigue Scores**	4](#_Toc207904698)

[**Supplementary Figure 4: Selected Smartwatch Peak Step Variables vs. Clinical Scores**	5](#_Toc207904699)

[**Supplementary Table 1: FLAIR Lesion Count**	6](#_Toc207904700)

[**Supplementary Table 2: Model Information for “Steps”**	6](#_Toc207904701)

[**Supplementary Table 3: Model Information for “Morning Steps”**	6](#_Toc207904702)

[**Supplementary Table 3: Model Information for “Afternoon Steps”**	7](#_Toc207904703)

[**Supplementary Table 5: Model Information for “Evening Steps”**	7](#_Toc207904704)

### **Supplementary Figure 1: Smartwatch adherence among study participants**

**Supplementary Figure 1: Smartwatch adherence among study participants.**

This figure illustrates the daily adherence of 24 patients to the smartwatch wear-time criteria during the study. One more patient opted for wearable monitoring, but no data was transmitted due to technical issues. The criteria required a minimum of 60% wear-time during wake hours (6 am - 11 pm). Days where the criteria were met are marked in blue, while days not meeting the criteria are shown in orange. Two patients who had less than 60% of days meeting the criteria (indicated by orange stars) were excluded from further analysis, one patient was excluded due to technical problems (orange diamond). The percentage of days meeting adherence criteria is written next to each patient’s respective bar at the right with median adherence across all patients at 98.8%.

### **Supplementary Figure 2: Selected Smartwatch Variables vs. Clinical Scores**

**Supplementary Figure 2: Selected Smartwatch Variables vs. Clinical Scores**

Heatmap illustrating Spearman correlations (ρ) between digital step count features (median daily, morning, afternoon, and evening steps) and clinical outcomes (EDSS, MSFC, 9HPT, T25FW, PASAT) in people with multiple sclerosis (pwMS). Positive correlations are shown in red, negative correlations in blue. Numbers represent correlations significant after Benjamini–Hochberg false discovery rate correction (q < 0.05); non-significant associations are left blank. Strongest associations were observed between evening and afternoon step activity with MSFC and 9HPT performance, while higher EDSS correlated negatively with evening steps.

### **Supplementary Figure 3: Selected Smartwatch Variables vs. Fatigue Scores**

**Supplementary Figure 3: Selected Smartwatch Variables vs. Clinical Scores**

Heatmap illustrating Spearman correlations (ρ) between digital step count features (median daily, morning, afternoon, and evening steps) and patient-reported fatigue measures (Worsening-of-Symptomps (WoS) Rate, FSMC, FSMC Motor, FSMC Cognitive, FSS, BFI) in people with multiple sclerosis (pwMS). Positive correlations are displayed in red, negative correlations in blue. Numbers would have only been shown for correlations significant after Benjamini–Hochberg false discovery rate correction (q < 0.05); non-significant correlations remain blank. However, after correction, no significant correlations remained.

### **Supplementary Figure 4: Selected Smartwatch Peak Step Variables vs. Clinical Scores**

**Supplementary Figure 4: Selected Smartwatch Peak Step Variables vs. Clinical Scores**

This figure illustrates correlations between step count-derived features from smwartwatches and clinical disability scores in people with multiple sclerosis (pwMS). Each dot represents one individual. Clinical disability was assessed using the Expanded Disability Status Scale (EDSS; panels A, C, E) and the Multiple Sclerosis Functional Composite (MSFC; panels B, D, F).

- (A–B) The relative spread between the 10th and 90th percentiles of hourly *peak* step counts - defined as the steps in the hour with the highest number of steps - normalized to the median peak steps per participant. No significant associations were observed with EDSS or MSFC.
- (C–D) Within participant normalized 10th percentile peak step counts (indirectly a measurement of variability as a lower score shows greater peak step variability), representing the lower bound of peak-hour activity, also showed no significant correlation with clinical outcomes.
- (E–F) The 90th percentile ratio of step counts in the morning (6-12 am) to afternoon (6-12 pm) hours, reflecting diurnal distribution of activity, did not correlate with either EDSS or MSFC.

Spearman correlation coefficients (ρ) and corresponding p-values are indicated in each panel.

### **Supplementary Table 1: FLAIR Lesion Count**

| **FLAIR Lesion Count** | **First MRI Scan** | **Second MRI Scan** |
| --- | --- | --- |
| >10 | 18 (70 %) | 18 (70 %) |
| >5 | 4 (15 %) | 4 (15 %) |
| >1 | 4 (15 %) | 4 (15 %) |

**Supplementary Table 1: FLAIR Lesion Count**

Table showing the number of FLAIR-hyperintense Lesions of the patients in the first and second MRI. The lesion count groups were stable between the first and second MRI

### **Supplementary Table 2: Model Information for “Steps”**

Boxplot Summary

| Segment | Mean | Std | N |
| --- | --- | --- | --- |
| Beginning | 0.08 | 0.13 | 21 |
| End | -0.02 | 0.21 | 21 |

OLS Regression Coefficients

| Variable | Coef | Std Err | t | p | CI [0.025] | CI [0.975] |
| --- | --- | --- | --- | --- | --- | --- |
| Intercept | 0.0980 | 0.048 | 2.035 | 0.042 | 0.004 | 0.192 |
| C(Cycle_Segment)[T.End] | -0.1317 | 0.047 | -2.833 | 0.005 | -0.223 | -0.041 |
| C(Cycle_Segment)[T.Middle] | -0.0845 | 0.047 | -1.797 | 0.072 | -0.177 | 0.008 |
| DiseaseDuration | -0.0004 | 0.002 | -0.241 | 0.809 | -0.004 | 0.003 |
| EDSS | -0.0062 | 0.013 | -0.484 | 0.628 | -0.031 | 0.019 |

OLS model: R² = 0.003, Adj. R² = 0.002, F(4,2737) = 2.131, p = 0.0745

### **Supplementary Table 3: Model Information for “Morning Steps”**

Boxplot Summary

| Segment | Mean | Std | N |
| --- | --- | --- | --- |
| Beginning | 0.07 | 0.21 | 21 |
| End | 0.04 | 0.16 | 21 |

OLS Regression Coefficients

| Variable | Coef | Std Err | t | p | CI [0.025] | CI [0.975] |
| --- | --- | --- | --- | --- | --- | --- |
| Intercept | 0.0237 | 0.048 | 0.494 | 0.622 | -0.070 | 0.118 |
| C(Cycle_Segment)[T.End] | -0.0015 | 0.046 | -0.032 | 0.975 | -0.092 | 0.089 |
| C(Cycle_Segment)[T.Middle] | -0.0813 | 0.047 | -1.742 | 0.082 | -0.173 | 0.010 |
| DiseaseDuration | 0.0008 | 0.002 | 0.442 | 0.659 | -0.003 | 0.004 |
| EDSS | 0.0017 | 0.013 | 0.131 | 0.896 | -0.023 | 0.027 |

OLS model: R² = 0.002, Adj. R² = 0.000, F(4,2726) = 1.103, p = 0.353

### **Supplementary Table 3: Model Information for “Afternoon Steps”**

Boxplot Summary

| Segment | Mean | Std | N |
| --- | --- | --- | --- |
| Beginning | 0.09 | 0.12 | 21 |
| End | -0.04 | 0.25 | 21 |

OLS Regression Coefficients

| Variable | Coef | Std Err | t | p | CI [0.025] | CI [0.975] |
| --- | --- | --- | --- | --- | --- | --- |
| Intercept | 0.0958 | 0.049 | 1.959 | 0.050 | -0.000 | 0.192 |
| C(Cycle_Segment)[T.End] | -0.1870 | 0.047 | -3.962 | 0.000 | -0.280 | -0.094 |
| C(Cycle_Segment)[T.Middle] | -0.0554 | 0.048 | -1.164 | 0.245 | -0.149 | 0.038 |
| DiseaseDuration | -0.0006 | 0.002 | -0.323 | 0.747 | -0.004 | 0.003 |
| EDSS | 0.0025 | 0.013 | 0.189 | 0.850 | -0.023 | 0.028 |

OLS model: R² = 0.006, Adj. R² = 0.005, F(4,2726) = 4.268, p = 0.0019

### **Supplementary Table 5: Model Information for “Evening Steps”**

Boxplot Summary

| Segment | Mean | Std | N |
| --- | --- | --- | --- |
| Beginning | 0.07 | 0.24 | 21 |
| End | -0.02 | 0.21 | 21 |

OLS Regression Coefficients

| Variable | Coef | Std Err | t | p | CI [0.025] | CI [0.975] |
| --- | --- | --- | --- | --- | --- | --- |
| Intercept | 0.0678 | 0.049 | 1.388 | 0.165 | -0.028 | 0.164 |
| C(Cycle_Segment)[T.End] | -0.1391 | 0.047 | -2.953 | 0.003 | -0.232 | -0.047 |
| C(Cycle_Segment)[T.Middle] | -0.0174 | 0.048 | -0.365 | 0.715 | -0.111 | 0.076 |
| DiseaseDuration | -0.0002 | 0.002 | -0.115 | 0.909 | -0.004 | 0.003 |
| EDSS | -0.0010 | 0.013 | -0.073 | 0.942 | -0.026 | 0.025 |

OLS model: R² = 0.004, Adj. R² = 0.002, F(4,2726) = 2.694, p = 0.0294
